# Stem cell treatment for regeneration of the rotator cuff: study protocol for a prospective single-center randomized controlled trial (Lipo-cuff)

**DOI:** 10.1186/s13063-024-08557-0

**Published:** 2024-10-19

**Authors:** Mariana Bichuette Cartuliares, Eva Kildall Hejbøl, Henrik Daa Schrøder, Andreas Kristian Pedersen, Lars Henrik Frich

**Affiliations:** 1 Department of Orthopaedics, Hospital Soenderjylland, Kresten Philipsens Vej 15, Aabenraa, 6200 Denmark; 2 Department of Regional Health Research, University of Southern Denmark, Campusvej 55, Odense M, 5230 Denmark; 3 Department of Regional Health Research and Institute of Molecular Medicine, Orthopaedic Research Unit, University of Southern Denmark, Campusvej 55, Odense M, 5230 Denmark; 4 Department of Pathology, Odense University Hospital, J. B. Winsløws Vej 15, Odense, 5000 Denmark; 5 Department of Clinical Research, University of Southern Denmark, Winsløwparken 19, Odense, 5000 Denmark; 6 Research Unit OPEN - Open Patient Data Explorative Network, J. B. Winsløws Vej 21, Odense, 5000 Denmark

**Keywords:** Rotator cuff tear, Stem cell treatment, Randomized controlled trial, Oxford shoulder score, Patient-reported outcome

## Abstract

**Background:**

Rotator cuff tears (RCT) are a common musculoskeletal condition, especially in the aging population. The prevalence of rotator cuff tears varies based on factors like age, occupation, and activity level. In the general population, the prevalence of rotator cuff tears is estimated to be around 20 to 25%. Rotator cuff tears (RCT) have an impact in patients’ pain level, shoulder function, sleep disturbance, and quality of life. Primary tendon surgery is in mostly cases necessary. This study aimed to examine if treatment of rotator cuff lesions with implantation of micro-fragmented adipose tissue can improve patients’ reported pain and function compared to conventional surgery.

**Methods:**

The study is a prospective superiority parallel-group single-center randomized controlled trial including 30 patients between 40 and 69 years of age in Denmark. Patients will be allocated 1:1 ratio to reconstruction of the supraspinatus tendon with an injection of micro-fragmented adipose tissue into the related muscle (stem cell treatment) or the standard of care (SOC), which is conventional surgery. Patients, project assistants, physicians, and outcome adjudicators are not blinded to randomization due to practical constraints. The radiologist and the statistician performing the analysis will be blinded. The primary outcome will be the Oxford shoulder score at 12 months post-surgery.

**Discussion:**

This study will assess whether adding micro-fragmented adipose tissue therapy to conventional rotator cuff tear treatment can enhance recovery, accelerate return to daily activities, and improve functional outcomes. The research will also determine if this minimally invasive procedure could be standardized for routine patient care.

**Trial registration:**

ClinicalTrials.gov NCT06505135. Registered on July 10, 2024.

**Supplementary Information:**

The online version contains supplementary material available at 10.1186/s13063-024-08557-0.

## Background

Rotator cuff tear (RCT) is a common shoulder disorder. It is estimated that 65–70% of all shoulder lesions are due to rotator cuff disease [1]. In the general population, the prevalence of rotator cuff tears is estimated to be around 20 to 25% [2]. According to data from the Danish National Patient Register, the incidence rate in 2013 was 715.3 per 10^5^ person-years at risk and the incidence was associated with an increase among people of working age [3]. The tears lead to weakness, pain, and reduced motility in the shoulder [4]. The patients experience markedly reduced quality of life, which also significantly burdens the healthcare system [5, 6], and premature withdrawal from the labor market or exclusion from the workforce is imminent [7]. The cause of rotator cuff tear is multifactorial. Acute trauma, as well as chronic stress, plays a part in combination with inherent factors [8, 9]. Therefore, patient experience is often incorporated into evaluating patient outcomes with a growing focus on standardized assessment and value-based healthcare, and an increased importance of patient-reported outcome measures (PROMs) that assess shoulder status after RCT [10].

Patient-reported outcomes such as Oxford shoulder score (OSS) and quality of life questionnaires are widely used in addition to clinical examination and imaging to assess shoulder status after RCT surgery [11, 12], focusing on value-based healthcare by quantifying a patient’s mental, physical, and functional health, offering a unique perspective to judge the success of surgery [10, 13].

When there is a total rupture of a tendon, surgery often fails to restore optimal function. Initial non-surgical treatment may provide temporary pain relief, but no healing of the lesioned tendon occurs [14]. Several studies indicate that degenerative changes in the damaged tendons and muscles could play a part in the suboptimal results of surgery [15–17].

A recent randomized study tested conventional primary tendon repair and physiotherapy in 103 patients. The authors reported significantly better results for primary tendon repair compared to conservative treatment. Normal function was not regained in any of the conservatively treated patients, and in this group, tear size increased over the years. Besides, patients who crossed to secondary surgery due to failed conservative treatment experienced significantly inferior results compared to primary surgery [18].

Therefore, a strategy that includes primary tendon surgery is meaningful, but with a need for additional treatment that restores the function of the damaged rotator cuff muscle.

Supraspinatus muscle biopsies from 33 patients treated at our hospital and undergoing rotator cuff repair demonstrated the presence of myogenic stem cells. We also found significant variations in stem cell activation, which seemed hampered according to patient age and also the age of the tendon lesion (Frick et al., manuscript submitted for publication, 2023).

Adipose-derived mesenchymal cells are shown to support regeneration in many tissues [19, 20], and animal studies [21], including our own (Frick et al., manuscript in preparation), indicate that injection of cultured adipose cells stimulates myogenesis by activating satellite cells in the torn supraspinatus muscle. The implantation of autologous micro-fragmented adipose tissue could, therefore, be a means for stimulating myogenesis, thereby improving the surgical treatment of rotator cuff lesions.

We hypothesize that combining surgical reconstruction with an injection of micro-fragmented adipose tissue into the supraspinatus muscle will be more effective for treating rotator cuff tears than conventional surgery alone. This approach is expected to result in superior patient-reported outcomes, clinical improvements, which will also be reflected in the radiological results.

The following objectives will be investigated:

1. To investigate if surgery of the shoulder muscle combined with implantation of micro-fragmented adipose tissue can improve function and reduce pain measured by the Oxford shoulder score at 1-year follow-up compared to conventional surgical treatment of rotator cuff lesions.
2. To investigate whether patients experienced improved shoulder function and pain, as measured by the Oxford shoulder score, at 3 and 6 months post-surgery, and enhanced quality of life, as measured by the EQ-5D-5L, at 3, 6, and 12 months post-surgery, following the combination of shoulder muscle repair and micro-fragmented adipose tissue implantation compared to conventional surgical treatment for rotator cuff lesions.
3. To examine if surgery of the shoulder muscle combined with implantation of micro-fragmented adipose tissue can improve clinical and radiological healing of the shoulder at 6 and 12 months compared to conventional surgical treatment of rotator cuff lesions.

## Methods

This protocol followed the recommended reporting guidelines for Standard Protocol Items: Recommendations for Interventional Trials (SPIRIT) [22]. The checklist for SPIRIT is attached as Additional file 1. The study will follow the Consolidation Standard of Reporting Trials (CONSORT) guidelines (parallel-group randomized trials) [23].

### Trial design

The study is a prospective superiority parallel-group single-center randomized controlled trial. The study will recruit patients with rotator cuff tear at the orthopedic outpatient clinic at the Hospital Sønderjylland in Sønderborg, a rural hospital servicing approx. 225.000 inhabitants, and a part of Denmark’s tax-funded healthcare system.

### Population and eligibility criteria

We will include patients between the ages of 40–69 years with clinical signs and symptoms compatible with a traumatic RCT, and who have an MRI verified supraspinatus tear and a reparable lesion with tendon retraction < 2 cm and fatty infiltration level 0–2 according to Fuchs et al. [24] and Goutallier classification [25]. Furthermore, based on the physical status classification system from the American Society of Anesthesiologists (ASA), patients have to be in good health (score < 3) [26].

#### Exclusion criteria

- Patients without an MRI of the shoulder
- Former surgery in the affected shoulder
- Signs of infection
- Immunosuppression (due to clinical condition or medical therapy)
- History of inflammatory disease
- Malignancy within 5 years
- Previous radiotherapy to the shoulder
- BMI under 18 or above 35
- Coagulopathy
- Non-Danish speaking patients

**Fig. 1:**
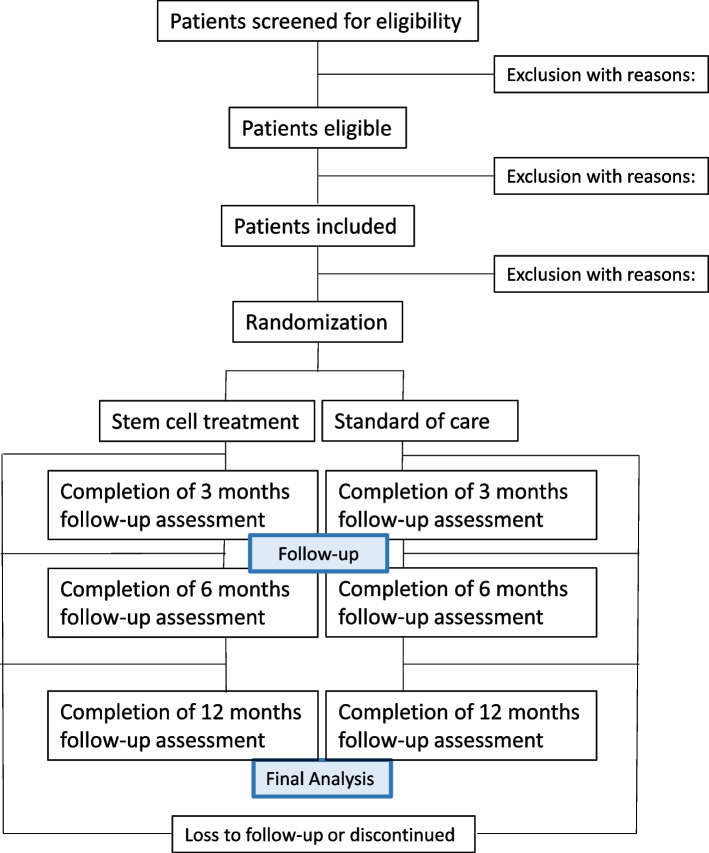
Trial population

These exclusion and inclusion criteria listed above are identical to those used for the conventional treatment except for the signed consent to the study and language clause (see Fig. 1).

#### Allocation

Patients will be allocated in a 1:1 ratio to receive either surgical reconstruction of the supraspinatus tendon with an injection of micro-fragmented adipose tissue into the related muscle (stem cell treatment) or the standard of care (SOC), which is conventional surgery.

Allocation will be administered by the project assistant or a physician and generated by a computer using a randomization sequence without stratification with permuting blocks, programmed in a separate document by an independent data manager with no role in the study. The randomization tool in the Research Electronic Data Capture (REDCap) will be used [27]. Patients will be assigned to either the stem cell treatment or SOC at the pre-surgery outpatient examination before their surgery appointment. Allocation concealment is ensured as the project assistant and the physician administering the randomization will be blinded to the randomization code, block sizes, and sequence throughout the trial. Additionally, the randomization is performed electronically and is only revealed after consent is obtained.

#### Blinding

Patients, project assistants, physicians, and outcome adjudicators are not blinded to randomization due to practical constraints. The radiologist and the statistician performing the analysis will be blinded.

### Interventions

After the clinical examination and obtained consent at the outpatient clinic, patients allocated to standard care or intervention groups will be scheduled for a shoulder surgery appointment.

#### Standard of care—rotator cuff surgery

Rotator cuff tendon suture is carried out at Hospital Sønderjylland according to the Danish guidelines for diagnosing and treating patients with shoulder disorders [14]. Standard treatment also includes intravenous injection preoperatively of 2 g of cloxacillin. In case of allergy, 1.5 g of cefuroxime is chosen. Tendon sutures are performed arthroscopically under regional analgetic blockage and light sedation using a standard double-row technique using suture anchors.

#### Stem cell treatment

Patients in the intervention group will receive a stem cell treatment added to the standard care rotator cuff surgery. Assisted by a plastic surgery, an expert in shoulder surgery will conduct all intervention procedures in cooperation with a team specialized in stem cell treatment.

##### Harvest of adipose tissue

Harvest of adipose tissue from abdominal subcutis using the Arthrex ACA Kit® (Arthrex GmbH, Naples, FL, USA), 30 ml of lipoaspirate will be collected from the lower abdomen. After skin preparation, the injection of stromal vascular fraction (SVF) will be performed on an outpatient basis under sterile conditions. In order to obtain the lipoaspirate necessary for the production of SVF, the patient will be injected with tumescent anesthesia and lipoaspirate will be removed from the abdominal fat tissue. Under sterile conditions, an infiltration of 150 ml tumescent solution consisting of 50 ml lidocaine, 1 ml epinephrine, 6 ml sodium hydrogen carbonate, and 1000 ml sodium chloride 0.9% will be performed.

ACP® double syringes. A Carraway Harvester® (Tulip Medical Products (San Diego, CA, USA)) will be connected to the syringes. The Arthrex ACP® double syringe consists of a large and a small syringe which is located in the plunger of the large syringe. The small syringe can be used to remove a liquid fraction that is above a solution after centrifugation without contaminating the remaining or the removed product. In the next step, the lipoaspirate, divided into 15 ml portions per double syringe, will be centrifuged at 2500 rpm in a centrifuge (Rotofix 32A® (Andreas Hettich GmbH & Co. KG, Tuttlingen, Germany) from Hettich Centrifuges) for 4 min at room temperature. The lipoaspirate will be divided into oil, fat graft, and an aqueous fraction. The oil will be transferred to the small syringe and discarded. The aqueous fraction will be removed. The fat graft will be transferred into two 10-ml Luer-Lock syringes and then transferred at least 30 times from one syringe to the other for homogenization using a 1.4-mm connector. Approximately 20 ml of fat graft per collection could be isolated and transferred 30 times from one syringe to the other for further processing.

##### Harvest of muscle biopsy from the supraspinatus muscle

A 0.1–0.2 g muscle biopsy is obtained from the supraspinatus muscle to estimate preoperative muscle fiber atrophy, intracellular lipid accumulation, mitochondrial dysfunction, inflammation, and reduced regenerative capacity. The muscle biopsy specimens will be taken during routine exposure or arthroscopy of the glenohumeral region. Using a blunt shaver, the RC and the musculotendinous junction of the supraspinatus muscle are gently debrided from the fascia and bursal tissue. Biopsies are snap-frozen on dry ice, stored at − 80 °C, or fixed in 10% neutral buffered formalin and embedded in paraffin.

The muscle biopsy will be analyzed with histology, including immunohistochemistry for fiber sizes, inflammation, fibrosis, adipocytes, and myogenesis.

##### Injection of micro-fragmented adipose tissue

In the cell treatment group of patients, injection of the cell suspension will be performed at the end of the surgical procedure. Fluid is carefully aspirated via the anterior outflow cannula, and autologous micro-fragmented adipose tissue is injected in dry arthroscopy conditions from the lateral portal while maintaining a subacromial view from the posterior portal.

Six milliliters of the stem cells suspension will be injected into the supraspinatus muscle at four predefined sites at the musculotendinous junction of the supraspinatus muscle. For each injection site, 1.5 ml of the suspension will be injected using an 18-gauge syringe.

##### In vitro control of the composition of the adipose tissue micro-fraction

The micro-fractions of adipose tissue not used for implantation will be used for control in 2 ways. Part of the material will be used to form a pellet, which can be fixed and embedded in paraffin. Sections of the pellet will be used to characterize the cell composition by immunohistochemistry for the expression of CD45, CD31, CD34, CD146, CD90, ASMA, and S100. These markers have previously been used to characterize adipose-derived cells [28]. Another part of the fractions will be used for explant cultures. In these cultures, the vitality of the cells can be inspected as out-growth from the explants and as proliferation capacity. Moreover, this material can also be used for immunohistochemical characterization of the cell types.

To improve adherence to intervention protocols, the surgical team have a plastic surgeon for the treatment.

#### Relevant concomitant care

All patients will be offered physiotherapy rehabilitation according to Danish clinical guidelines [14].

## Outcomes

### Primary outcome

#### The Oxford shoulder score (OSS) questionnaire

The primary endpoint is the patient-reported shoulder function assessed by the Oxford shoulder score 12 months after surgery reported in mean [11]. The OSS comprises 12 items: four assessing the degree of pain and eight evaluating function. Each item is rated on a 5-point Likert scale, where 0 indicates the worst outcome, and 4 indicates the best. The scores from these 12 items are summed to produce a total score ranging from 0 to 48 [11].

### Secondary outcomes

#### The Oxford shoulder score questionnaire

Improvements in the Oxford shoulder score as above [11] measured 3 and 6 months post-surgery compared to baseline.

#### The EuroQol-5 Dimension (EQ-5D-5L) questionnaire

Median improvements from the baseline in the EQ-5D-5L including self-rated health measured by a visual analog scale (VAS) [29] were assessed at 3, 6, and 12 months, with the 12-month measurement being of primary importance. The EQ5D measures the quality of life on a 5-point Likert scale based on mobility, self-care, usual activities, pain/discomfort, and anxiety/depression, ranging from “no problems” to “extreme problems” [30, 31]. A key feature of the EQ-5D is the availability of “value sets” that weight the health states reported by patients into utility indexes according to the preferences of a country. For the Danish population, these values range from − 0.757 to 1.0, where 1.0 corresponds to absolute health, 0 corresponds to death, and negative values correspond to a health status considered worse than death [32]. The VAS scale is numbered from 0 to 100. 100 means the best health you can imagine. 0 means the worst health you can imagine [29].

#### Clinical healing

Clinical healing will measure pain, function, and strength. (1) Pain will be documented in a range from 0 to 10 and reported in median, (2) pain-free movement (anyhow) above shoulder level reported as yes/no. If the patient is able to have the arm above the shoulder without pain, a (3) strength test will be performed with ISOBEX 4.0, CH3482 Schweiz, and a median for three tests will be recorded in newton. Clinical assessment will be performed at 3, 6, and 12 months in the outpatient clinic and compared between intervention and standard care groups.

#### Radiological healing

This will be defined as the closure of the gap between the tendon and the greater tuberosity that serves as the attachment for rotator cuff tendon, as assessed by magnetic resonance imaging (MRI). MRI provides important static information on muscle atrophy and fatty infiltration of muscle, of which the latter is closely related to the integrity of the repaired tendon and postoperative functional outcomes and is a known important prognostic factor in rotator cuff tear [25]. Muscle changes will be followed using magnetic resonance imaging (Dixon method) [33] 6 and 12 months after surgery. Changes between baseline MRI, 6 and 12 months will be reported and compared between groups. MRI will give information on (1) supraspinatus tendon healing (yes/no) and if the tendon is healed categorized as not healed, partially healed, or completely healed, reported as numbers and percentages, (2) cross-section area supraspinatus on Y in mm^2^, (3) the adjusted cross-section area supraspinatus on Y in mm.^2^, (4) supraspinatus muscle belly atrophy will be evaluated according to Warner et al. (categorized as 1—none atrophy, the muscle completely fills its fossa, and the outer contour is convex, 2—minimal atrophy = muscle’s outer contour is flat compared with its fossa, 3—moderate atrophy = muscle’s outer contour is concave into the fossa, 4—severe atrophy = muscle is barely apparent in its fossa) [34], (5) fatty degeneration will be categorized in three levels according to Fuchs et al. (0—normal muscle or some fatty streaks, 1—more fat than muscle, or 2—equal or greater amount of muscle than fat) [24], and (6) after Goutallier classification (grade 0—normal muscle without fat, grade 1—some fatty streaks within the muscle, grade 2—less fat than muscle within the muscle, grade 3—same amount of fat and muscle within the muscle, and grade 4—more fat than muscle within the muscle) [35]

#### Functioning of supraspinatus muscle

Improvement of muscle strain in the supraspinatus muscle recorded by speckle tracking ultrasonography (STU) [36]. Muscle strain is a quantitative measure of the contractile properties of muscle tissue [37], and STU patterns will be used to quantify changes in muscle contractility for direct recording of muscle recovery. The maximum voluntary contractions will be reported in newton and the median of five measures of muscle strain of 20%, 40%, and 60% will be documented. The examination will stop if pain is reported. STU will be performed 3, 6, and 12 months after surgery, and changes between the time points will be registered and compared between groups.

### Participant timeline

#### Recruitment

**Fig. 2:**
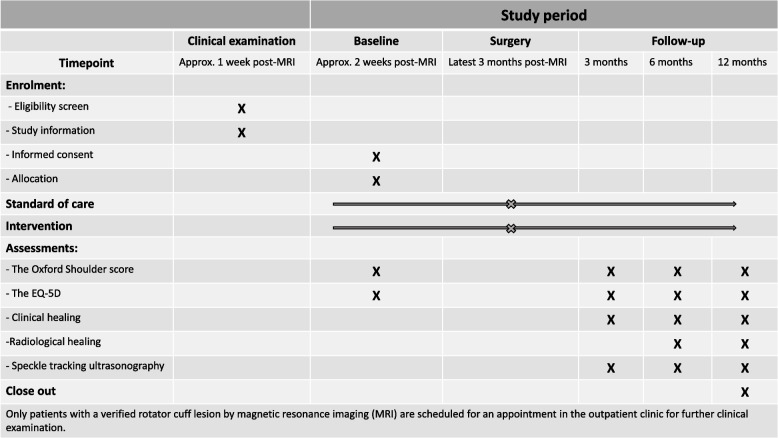
SPIRIT figure with the schematic diagram for the schedule for the participants

Patients with a confirmed rotator cuff lesion verified by an MRI will have an appointment at the outpatient clinic, where a physician will perform a clinical examination and screening for the study. The responsible surgeon will inform and provide instructions to the physicians performing the first clinical examination and screening of the patients to ensure all patients will be screened. Patients who fulfill the inclusion and exclusion criteria will receive written and oral information about the study provided by the responsible surgeon. As required by ethical rules in Denmark, a quiet atmosphere, a consideration period of a minimum of 24 h, and the possibility of having a companion will be ensured. Consent will be signed by the patient and the surgeon responsible for the study during a subsequent visit. Only patients who give informed consent will be participating in the study. The timeline for the schedule for the participants is presented in Fig. 2.

### Data collection, management, and analyses

#### Data collection methods

To promote data quality, ensure consistency, reduce the amount of missing data, and increase responsiveness, the project assistant will instruct the study personnel in data collection and reporting. Furthermore, the project assistant will check and supplement for missing data if possible. The patients will fill out the OSS and EQ-5D on their phones in the waiting room at the outpatient clinic, and a study assistant will be present to ensure the successful management of the technology.

All data will be collected through patient interviews regardless of the OSS and EQ-5D questionnaires, which the patients will complete independently.

The demographic data will include patients’ age, gender, social status, occupation, and education. Lifestyle variables comprise smoking history (no, a current smoker, and yes with the amount), alcohol consumption (under or over ten doses), and physical activity (none, under or over 2.5 h). The alcohol consumption report follows the Danish National recommendations [38], and physical activity follows recommendations from the World Health Organization [39]. Clinical data reported will include lesion age in days, how the patient got injured, and sick leave. Pain will be reported on a scale from 0 to 10 at each clinical examination. Medicine information comprises if patients have an intake of statins, any painkillers, or have multipharmacy (> 5 medications). Adverse events will be registered with a date and a description.

#### Data management

All data will be entered electronically in REDCap, a secure software data management platform [27]. To promote data quality, all variable coding has been consistently planned with required fields to reduce missing data, field notes explaining how to register a variable and validation for numerical variables. An independent data manager has double-checked all instruments in REDCap. Furthermore, only the project assistant and the independent project manager can access the database’s design and development. The others collecting data will only have the possibility to register in the database. It secures data quality and reduces bias.

### Statistical methods

#### Sample size

To calculate the sample size, we used Monte Carlo simulation. We based the simulation on a linear regression. Hence, the estimated sample size from the Monte Carlo simulation is a conservative estimate of the needed sample size, as we will utilize a mixed effect model in the primary analysis. In the simulation, we assumed the mean score and its standard deviation were distributed across different time points, as highlighted in Table 1, similar to the results reported by Cartuliares et al. (manuscript submitted for publication, 2024) derived from a prospective cohort including patients who underwent rotator cuff surgery at the Hospital of Sønderjylland. This study, along with a clinical assessment by experts in shoulder surgery and stem cell therapy, determined that an 8-point increase in the OSS, equivalent to a 25% improvement, will be accepted as the minimal clinically significant difference, forming a background for the power calculation. The Do-File from STATA/BE used for the calculation is in Additional file 2.

**Table 1:** Data used for the power calculation

Time point	Baseline	3 months	6 months	12 months
Mean score (SD) control group	19 (4.12)	28 (4.12)	30 (4.12)	33 (4.12)
Mean score (SD) intervention group	19 (4.12)	40 (3)	36 (3.6)	41 (3.6)

*SD* Standard deviation

The *p* value was based on the difference at 12 months, where we adjusted for multiple testing through Bonferroni correction. Under these assumptions, we needed 30 patients in total (15 in each arm) to achieve a power of 94% at an alpha level of 0.05.

#### Statistical analyses

Descriptive statistics will be applied to check for exchangeability between the two groups according to the variables measured at baseline and to assess the reproducibility of our sample with previous study within this field. Non-categorical data will be presented as mean and standard deviation (SD) and analyzed using a *t*-test or median and interquartile ranges (IQR) analyzed with Wilcoxon rank sum test, depending on whether the given variable follows a normal distribution. Depending on Cochran’s rule, categorical data will be presented as numbers (*n*) or proportions (%) and analyzed with either a Fisher or chi-square test, depending on Cochran’s rule.

Besides the descriptive statistics, margin plots for the primary and secondary outcomes will be constructed to assess whether the treatment effect changes over time.

The final manuscript will report 95% confidence intervals and two-sided *p* values. A *p* value below 0.05 will be considered statistically significant and multiple testing will be handled by using Hochberg’s sequential procedure. All data will be analyzed in STATA/BE version 18.

##### Primary analysis

The primary analysis will be a beta-binomial mixed effect model, thus adopting a subject-specific approach. We will incorporate an interaction between group and time if necessary, to accommodate varying treatment effects over the time period. Therefore, we will present the estimates for each time point (baseline, at 3, 6, and 12 months) and construct a margin plot based on the model. We will conduct a likelihood ratio test to test whether our intervention affects the primary outcome across all time points. If the test is statistically significant, we will conduct a *z*-test at each time point to assess when there is a statistically significant treatment effect. Here, we will utilize G-computation to acquire interpretable effect sizes on both the mean ratio and mean difference scales for sensitivity purposes. In the G-computation, the Q-model will be a mixed-effect model, and the prediction will be based solely on the fixed effect. To avoid inflating the type I error rate in the bootstrapped *z*-test, we will adjust the *z*-test for multiple tests.

If the beta-binomial regression has difficulties converging, we will adopt a mixed-effect model with bootstrapped confidence intervals. Here, we will choose restricted maximum likelihood and the Kenward method for calculating the degrees of freedom and test whether a random slope shall be incorporated by way of an *F*-test.

The primary analysis will follow the intention-to-treat principle; hence, missing data will be managed. As the beta-binomial mixed effect model regression uses maximum likelihood estimation, we can assume that the beta regression will yield unbiased results if the data is missing at random or completely at random. However, under the assumption of missing not at random, the estimates from the beta regression will be biased; therefore, we will conduct joined modeling to assess the influence of missing data if the data was missing not at random. In the joint model, we will use Cox regression to estimate the censoring weights.

##### Secondary analysis

The analysis of EQ-5D will follow the same principle as the primary outcome. Generalized linear mixed-effect models will be chosen for all secondary outcomes and utilize the same parameterization as the primary outcome analysis. For EQ-5D-VAS, pain, supraspinatus healing, and fatty degeneration, we will use a mixed-effect beta-binomial regression, and if convergence fails, we will perform a mixed-effect linear regression with bootstrapped 95% confidence intervals (CI). Concerning the outcomes measuring pain-free movement above the shoulder (yes/no) and supraspinatus atrophy (yes/no), we will use a mixed-effect logistic model. For outcomes relating to MRI and STU assessment, we will utilize a mixed-effect model. If the model fit for the mixed-effect model is deemed unacceptable. First, a log transformation of the outcome will be utilized, and if the fit is still unsatisfactory, we will use a mixed-effect gamma regression.

If any regression analyses produce problematic effect sizes, we will use G-computation, assuming the Q-model is the appropriate generalized linear model analysis for the given outcome. Additionally, we will account for the longitudinal aspect using multiplicative interaction and baseline restriction.

Interim analysis only concerning adverse events will be performed for every 5 patients included, and early trial cessation is possible if there are any safety concerns. This analysis will be presented descriptively.

Sensitivity analyses will be conducted to compare the standard care and intervention groups across each domain of the OSS and EQ-5D. These analyses will track changes in each domain’s response trajectory from baseline to 3, 6, and 12 months post-surgery. The results will be descriptively analyzed and graphically presented using radar plots.

To enhance retention and minimize loss to follow-up, we will have an automated electronic system for scheduling and reminders for upcoming visits at the outpatient clinic. Additionally, we will offer flexible appointment times to better accommodate participants’ schedules. We will continuously monitor retention rates and proactively reach out to patients in the event of missed appointments.

### Monitoring

The project assistant responsible for managing and developing the study database will monitor data collection to ensure minimal missing data. The project assistant has no role in screening, the surgery, or allocation performance but will assist in clinical assessment if needed, such as collecting demographic information from patients and performing STU. The project assistant will monitor the daily inclusion of participants and discuss with the project group.

After the primary data analysis, the results will be discussed and evaluated by the project group and all involved, including the hospital department.

### Adverse events, complications, and risks

The liposuction procedure is safe with a very low risk of complications (less than 1%), such as bleeding, infections, and scarring [40]. In rare cases, there may be bleeding, and a reoperation or puncture of the skin may be necessary. An infection is rarely seen and can be treated with antibiotics, and in exceptional cases, additional surgery may be required. Sensory disturbances might be seen at the donor site. These fade away generally within 3–6 months, but in some individuals, it may be permanent (less than 1%).

Harvest of 0.1–0.2 g muscle biopsies from the supraspinatus muscle is performed under direct vision from the arthroscope and has been performed without complications in 42 patients in a former protocol (Projekt-ID: S-20160037).

MRI and ultrasonography have no reported risks or adverse effects.

The responsible surgeon or physician will report adverse events in the study database at the time they occur. All adverse events occurring during surgery, post-surgery, and up to the final follow-up at 1 year will be documented. Adverse events that occur after a subject has been discontinued from the study will not be reported unless the investigators suspect they are related to the stem cell treatment procedures. We will record adverse events and any negative effects of the intervention using a structured template that allows for additional comments in free text within the electronic database (REDCap). For each event, we will document the date, type of adverse event, or negative effect (e.g., infection, skin and subcutaneous tissue disorders, increased pain, reduced strength, reduced mobility, reduced sensitivity, or other issues). Any identified harms will be detailed in future publications.

#### Reasons for early study termination

Continued analysis for safety will be performed regarding the presence of major adverse events, and an interim analysis will be formed after every five patients are included. Major adverse events are defined as postoperative complications requiring active treatment (the Clavien-Dindo classification grade III and above). In case of exclusion, patients are offered standard treatment according to hospital standards. The study will be terminated early if this analysis reveals the presence of major adverse events in 50% or more in one arm of the study. The Danish Patient Compensation covers patients enrolled in this study.

Only the project assistant who developed the study database and monitors the data will have access to the interim analysis. This assistant will extract a predefined report containing only reported adverse events, ensuring that all other data remains confidential. If adverse events are identified, they will be presented to the study group, including the principal investigator, and an audit will be performed before deciding whether to terminate the trial. There will not be a possibility of modifying the intervention, but patients will receive standard treatment.

## Ethics and dissemination

### Research ethics approval

The study protocol, version 8, was approved by the Regional Committees on Health Research Ethics for Southern Denmark on June 27, 2024 (S-20230073), registered by the Danish Data Protection Agency (Journal nr.: 24/26916) and by the ClinicalTrials.gov (NCT06505135).

The study will be conducted according to the study protocol, which conforms to the current health legislations and good clinical practice (ICH-GCP), and written informed consent will be obtained from the participants before participation in the study (Additional file 3). Participation is voluntary, and patients may withdraw their consent at any time without consequences for further treatment. The data collected from discontinued patients will be deleted and will not be part of the analysis.

To protect patient confidentiality, the personal data collection will be registered in the REDCap electronic database [27], data extraction for data analysis will be pseudonymized, and the results data will be presented anonymously. The patient will be informed both verbally and in writing that the tissue sample will be stored in a research biobank at the Department of Pathology in Odense, along with details about any future analyses. Participants will have no financial gain by participating in the study. The advantage for the patients may be improved rehabilitation and reduced risk of fibrosis. Stem cell therapy has, however, been shown to be safe. Intra-articular injection of the lipoaspirate is formerly approved to be used at Hvidovre Hospital (Danish National Committee on Health Research Ethics (H-18013145) and the Danish Data Protection Agency (VD-2018–141)). This study can potentially establish the fundaments of a safe treatment for an otherwise difficult surgical condition.

### Protocol amendments

Any important protocol modifications will be directly communicated to cooperators, registered in the national clinical trials register and ethical committee, and be clearly stated in future publications.

### Ancillary and post-trial care

The procedures are known to have minimal risks and the hospital will provide post-trial care as routinely.

### Dissemination policy

The results whether negative or positive will be presented to all stakeholders and will be published in an open reviewed journal. Substantive contributions to the design, execution, interpretation, and reporting of the clinical trial will be acknowledged with authorship on the final trial report, in accordance with the guidelines of the International Committee of Medical Journal Editors (ICMJE).

### Biological material storage biobank

During the study period, acquired biological materials from participating patients will be registered and stored in the research biobank at the Department of Clinical Pathology, OUH, according to current legislations and requirements. The materials include aliquots of freshly isolated micro-fragmented adipose tissue products and processed products hereof to enable analyses and comparisons with clinical efficacy. After the study is completed, stored materials are transferred to a biobank for future research. Future research will involve analyses of the cellular content, with the aim of comparing the cell composition to the treatment outcomes and to investigate how freezing affects fat-derived stem cells, with a focus on potential phenotypic changes in their differentiation capacity.

## Discussion

Treatment of rotator cuff tears with micro-fragmented adipose tissue is a minimal-invasive procedure with the potential to shorten and ease recovery, accelerate return to daily activity and work of thus with a potential capacity to improve the functional result compared to conventional surgery alone.

The study will provide evidence whether addition of micro-fragmented adipose tissue therapy can augment conventional rotator cuff tear treatment. The study will also reveal whether this treatment can be feasible for standard care of patients with rotator cuff tear as it will be simple to standardize. Moreover, besides providing a novel treatment for patients with rotator cuff tears, the project will based on data from muscle biopsies and scanning modalities generate new knowledge, preparing for precision regenerative medicine in shoulder disease. We expect that we after the project period will be able to initiate a phase 2 clinical trial to further implement precision medicine as a standard treatment for patients suffering rotator cuff tear. The results of the study will have both national and international interest, as this stem cell treatment can easily be applied in hospitals with a similar context and resources.

## Trial status

ClinicalTrials.gov: NCT06505135.

Protocol version number 8. Date: 22.06.2024.

Date start of recruitment: September 1, 2024.

Approximate date recruitment to be completed: approx. December 2025.

## Supplementary Information

Additional file 1.

Additional file 2.

Additional file 3.

## Data Availability

Due to Danish laws on personal data, data cannot be shared publicly. To request these data, the corresponding author must be contacted for more information. The person responsible for the research was the principal investigator and corresponding author (LHF) who together with the Department of Health Research and the University Hospital of Southern Denmark, owns the data and has access to the final data set.

## Abbreviations

PROM: Patient-reported outcome measure
RCT: Rotator cuff tear
