# Supplementary material for: Stem cell treatment for regeneration of the rotator cuff: study protocol for a prospective single-center randomized controlled trial (Lipo-cuff)
Source: Trials. 2024 Oct 19;25:696. doi: 10.1186/s13063-024-08557-0 (PMC11492208; doi:10.1186/s13063-024-08557-0)
Supplement: Supplementary file 2 — Additional file 2. [file 13063_2024_8557_MOESM2_ESM.pdf]

Kopi of STATA do-file for the power calculation

```
clear
local mc = 1000
set obs `mc'
generate data_store = .
generate power=0
generate Power=.
quietly {
forvalues i=1(1)`mc' {
if `i'==100|`i'==200|`i'==300|`i'==400|`i'==500|`i'==600|`i'==700|`i'==800|`i'==900{
noisily display `i'/`mc'*100 "% done"
}
preserve
clear
//set seed `i'
set obs 30
gen id=_n
expand 4
gen treatment=rbinomial(1,0.5)
bysort id: gen time=_n
gen outcome=rnormal(19,4.12)
replace outcome=rnormal(5.67+19,3) if time==1
replace outcome=rnormal(9.96+19,3) if time==2
replace outcome=rnormal(12.82+19,3.6) if time==3
replace outcome=rnormal(19+8,4.12) if time==3 & treatment==1
replace outcome=rnormal(19+6,4.12) if time==1 & treatment==1
replace outcome=rnormal(19+6,4.12) if time==2 & treatment==1
gen missing=0
replace missing=rbinomial(1,0.05) if time==2
replace missing=rbinomial(1,0.08) if time==3
```

```
regress outcome i.treatment##i.time, vce(bootstrap)

estimates store m1

regress outcome i.time, vce(bootstrap)

estimates store m0

bootstrap p=r(p): lrtest m1 m0

matrix define A=r(table)

restore

replace data_store = A[1,1] in `i'

replace power=1 if data_store<0.025

replace Power=sum(power)

}

/*Monte Carlo simulationen ville have konvergeret hvis kurven flader ud*/

generate iteration=_n

replace Power=Power/iteration

}

twoway (connected Power iteration)

tab power
```
