# Supplementary material for: Stem cell treatment for regeneration of the rotator cuff: study protocol for a prospective single-center randomized controlled trial (Lipo-cuff)
Source: Trials. 2024 Oct 19;25:696. doi: 10.1186/s13063-024-08557-0 (PMC11492208; doi:10.1186/s13063-024-08557-0)
Supplement: Supplementary file 3 — Additional file 3. [file 13063_2024_8557_MOESM3_ESM.pdf]

(S4)

Informeret samtykke til deltagelse i et sundhedsvidenskabeligt forskningsprojekt.

Forskningsprojektets titel: *Stamcelle behandling af sygdomme i skulderens senemanchet*

Erklæring fra forsøgspersonen:

Jeg har fået skriftlig og mundtlig information og jeg ved nok om formål, metode, fordele og ulemper til at sige ja til at deltage.

Jeg ved, at det er frivilligt at deltage, og at jeg altid kan trække mit samtykke tilbage uden at miste mine nuværende eller fremtidige rettigheder til behandling.

Jeg giver samtykke til, at deltage i forskningsprojektet og til, at mit biologiske materiale udtages med henblik på opbevaring i en forskningsbiobank. Jeg har fået en kopi af dette samtykkeark samt en kopi af den skriftlige information om projektet til eget brug.

Forsøgspersonens navn: ____

Dato: ________________ Underskrift: ____

Hvis der kommer nye væsentlige helbredsoplysninger frem om dig i forskningsprojektet vil du blive informeret. Vil du **frabede** dig information om nye væsentlige helbredsoplysninger, som kommer frem i forskningsprojektet, bedes du markere her: ___________ (sæt x)

Ønsker du at blive informeret om forskningsprojektets resultat samt eventuelle konsekvenser for dig?:

Ja _____ (sæt x) Nej _____ (sæt x)

Erklæring fra den, der afgiver information:

Jeg erklærer, at forsøgspersonen har modtaget mundtlig og skriftlig information om forsøget.

Efter min overbevisning er der givet tilstrækkelig information til, at der kan træffes beslutning om deltagelse i forsøget.

Navnet på den, der har afgivet information: Lars Henrik Frich

Dato: ________________ Underskrift: ____

Projektidentifikation:Komiteens Projekt-ID: S-2023-0073

Projekt til behandling af sygdomme i skulderens senemanchet

Deltagerinformation om deltagelse i et videnskabeligt forsøg

Forskningsprojektet handler om stamcellebehandling af sygdomme i skulderens senemanchet
Original titel: Stem cell treatment for regeneration of the rotator cuff (the Lipo-Cuff Study)

Tak for din interesse i projektet ”Stamcelle behandling af sygdomme i skulderens senemanchet”.
I forbindelse med udredning af din skulderlidelse er du blevet tilbudt en operation med syning af din overrevne senemanchet (rotator cuff). Vi vil i den forbindelse spørge dig om du vil deltage i et forsøg hvor din behandling suppleres med celleinjektion. Behandlingen er eksperimentel og skal undersøge om stamceller kan forbedre mulighederne for bedre heling og dermed hurtigere genoptræning af skulderen.
Før du beslutter, om du vil deltage i forsøget, skal du fuldt ud forstå, hvad forsøget går ud på, og hvorfor vi gennemfører forsøget.

Baggrund

Sygdomme i senerne i skulderens rotator cuff er hyppigt forekommende og ca. en tredjedel af alle danskere vil med tiden udvikle symptomer fra skulderen af denne årsag. Skader på rotator cuffen er hyppige i 40-60 års alderen blandt arbejdsduelige personer og i alvorlige tilfælde kan der ske overrivning af en eller flere sener (rotator cuff læsion).
Skaderne kan være traumatiske, dvs. opstået i en sund sene efter en større enkeltstående påvirkning (fx ved at man falder) eller degenerative, dvs. opstået uden nogen særlig fysisk påvirkning i en sene, som på forhånd er svækket. De fleste rotator cuff skader har både et traumatisk og et degenerativt aspekt. Kun patienter med relativt friske rotator læsioner tilbydes operation.
Skader på rotator cuffen er forbundet med smerter, nedsat funktion i skulderen og dermed tab af livskvalitet og arbejdsevne. Behandling med fysioterapi kan have en midlertidig gavnlig effekt på smerter, men de beskadigede sener i skulderen heler ikke spontant. Heller ikke kirurgisk behandling med påsyning af de overrevne sener er i dag optimal. I mange tilfælde genvindes skulderfunktionen kun delvist, hvilket i høj grad påvirker patientens samlede liv, herunder adgangen til arbejdsmarkedet.
Forskning, der kan forbedre reel heling af skader på rotator cuffen, er således nødvendig. Siden 2016 har vores forskningsteam undersøgt patienter, der alle er kirurgisk behandlet for rotator cuff

læsion. Vi har hos disse patienter fundet forandringer i muskulaturen, der delvis erstattes af fedt- og bindevæv. Disse forandringer forhindrer normal skulderfunktion. Resultaterne af operation afhænger derfor af både senens og musklens heling.

Formålet med forsøget

Vi har i laboratoriet analyseret muskelprøver fra patienter med rotator cuff overrivning. Prøverne viser, at de skadede muskler indeholder mange stamceller (satellit-celler) i dvaletilstand. Vores dyreforsøg har vist, at helingspotentialet i musklen forbedres og at betændelses- og bindevævsdannelsen reduceres ved tilførsel af stamceller til de dvale-liggende satellitceller. Vi er nu klar til at afprøve, hvorvidt stamcellebehandling kan genskabe og reparere muskelfibre i den skadede muskel ved patienter, der skal opereres for rotator cuff læsion.

Plan for forsøget

Patienter indstillet til operation for rotator cuff skade på Sygehus Sønderjylland vil blive spurgt om deltagelse i undersøgelsen indtil 52 patienter har accepteret deltagelse.

Det er en forudsætning for deltagelse i forsøget at seneskaden er bekræftet ved en MR skanning og at alle patienter har deltaget i et extraordinært informationsmøde om forsøget.

Lodtrækning udføres under informationsmødet, hvorefter du får at vide hvilken behandling du vil modtage. 26 patienter vil få standard behandling uden tilførsel af stamceller. I lodtræknings forsøgets interventionsgruppe, får patienterne syet den overrevne rotator cuff ved den almindelige standardoperation. Derefter behandles de med stamceller, udvundet fra eget fedtvæv i maveregionen. Forsøgets kontrolgruppe behandles med konventionel kirurgi uden tilførsel af stamceller. Lodtrækningen sker umiddelbart efter din underskift og accept af deltagelse i forsøget. Resultaterne af forsøget monitoreres på flere forskellige måder.

Hvad indebærer deltagelse

I forbindelse med din skulderoperation på ortopædkirurgisk afdeling vil vi samtidig udtage en vævsprøve på ca. 0,1 g fra skuldermusklen ved den overrevne sene. Muskelprøven bliver anvendt til at vurdere eventuelle skader i vævet. Muskelvævet gendannes som regel i løbet af få uger.

Hvis du kommer i behandlingsgruppen udtages ca. 150 ml fedtvæv fra maveregionen ved en fedtsugning. Fedtvævet homogeniseres og koncentreres til en opløsning på 10 ml, som efterfølgende injiceres i skuldermusklen tæt på senens tilhæftning.

Din vævsprøve vil være placeret i en forskningsbiobank på Patologisk afdeling i Odense. Prøven anonymiseres efter studiets afslutning. Der indhentes efter din indskrivelse i projektet relevante helbredsmæssige oplysninger fra din journal til brug i undersøgelsen. Disse oplysninger omfatter din alder, hvor gammel din skade er, BMI, alkohol og tobaksforbrug samt brug af smertestillende medicin. Alle faktorer, som kan have betydning for helingsprocessen.

Deltagere i forsøget vil blive undersøgt i alt 4 gange efter forundersøgelse i dag. Disse kontroller foregår hhv. 1, 3, 6 og 12 måneder efter operationen. Der er således én ekstra kontrol (ved 6 og 12 måneder) i forhold til standardbehandlingen.

Vigtigst er din egen bedømmelser af operationsresultatet. Du vil derfor i forsøgsperioden blive bedt om elektronisk at udfylde et spørgeskema (Oxford Skulder Score) som gør det muligt at vurdere din værdi af operation og eventuel tilførsel af stamceller. Vi anser en forbedring på 20%, hvilket er lig med en 8 point forbedring i den danske version af Oxford Shoulder Score (0-48 points, hvor 48 er max) for en vellykket behandling.

Den kliniske effekt af stamcellebehandlingen monitoreres også gennem ultralydsskanning hhv. 1, 3, 6 og 12 måneder efter operationen samt ved MR skanning efter 6 og 12 måneder. MR skanning og ultralydsskanning indgår i Sygehus Sønderjyllands standardkontroller, men skanningerne efter 6 og 12 måneder efter operationen er også en del af forsøget. Hverken ultralyds eller MR skanninger er forbundet med bivirkninger

Biologisk materiale

De overskydende 1-5 ml af fedtopløsningen bruges til analyser af celleindholdet med henblik på at sammenligne cellesammensætning med behandlingsresultatet. Noget af cellematerialet fra fedtsugningen skal også bruges til udarbejdelse af isolerings- og dyrkningsprotokoller til fremstilling af adskilte celletyper. Materialet vil blive registreret og opbevaret i en forsknings biobank på Afdeling for Klinisk Patologi, Odense Universitets Hospital. Efter forsøgets ophør vil prøverne blive opbevaret i samme biobank til eventuel fremtidig forskning.

Et nyt forskningsprojekt vil blive anmeldt til den videnskabsetiske komité. I tilfælde af ny forskning vil du blive bedt om et nyt samtykke, med mindre komiteen finder det rimeligt at give dispensation for dette. I hele forløbet vil databeskyttelsesreglerne fortsat overholdes.

Etik og rettigheder for forsøgspersoner

Du vil inden operationen blive inviteret til en samtale om forsøget og hvor denne deltagerinformation vil blive uddybet og hvor du kan stille spørgsmålet til forsøget. Du er

velkommen til at tage en pårørende eller ven med. Vi beder dig også om at læse deltagerinformation samt ”Forsøgspersoners rettigheder i et sundhedsvidenskabeligt forskningsprojekt”.
Hvis du beslutter dig for at deltage i forsøget vil vi bede dig om at underskrive en samtykkeerklæring. Husk at du har ret til betænkningstid før du underskriver erklæringen.
Det er frivilligt at deltage i forsøget. Du kan når som helst og uden at give en grund trække dit samtykke tilbage. Dit behandlingsforløb vil ikke blive påvirket af din beslutning.

Nytte ved forsøget
Projektet vil bidrage til at udvikle en behandling, der sikrer en bedre skulderfunktion for den enkelte patient efter operation for rotator cuff læsion. Det vil hjælpe til en bedre identifikation af de patienter der kan have gavn af stamcellebehandling. Projektet vil desuden bidrage med øget viden om behandling af patienter med rotator cuff læsion.

Bivirkninger, risiko, komplikationer og ulemper ved forsøget
Deltagelse i undersøgelsen er ikke forbundet med yderligere bivirkninger end de bivirkninger, som er forbundet med din skulderoperation. Disse bivirkninger er omfattet af patienterstatnings ordningen.
Der er ingen kendte bivirkninger ved stamcelle-transplantationen, da det er dine egne celler, der anvendes. Der kan imidlertid være andre risici ved forsøget, som vi endnu ikke kender. Vi beder dig derfor om at fortælle, hvis du oplever problemer med dit helbred, mens forsøget står på. Hvis vi opdager bivirkninger, som vi ikke allerede har fortalt dig om, vil du naturligvis blive orienteret med det samme, og du vil skulle tage stilling til, om du ønsker at fortsætte i forsøget.
Udtagelse af fedtvæv fra din maveregion er forbundet med få komplikationer og mindre end 1% oplever mindre komplikationer. I sjældne tilfælde kan der derfor være behov for at udtømme en blodansamling. Infektioner er også sjældne og kan som hovedregel behandles med antibiotika. Føleforstyrrelser omkring arret på maven kan forekomme. Disse forsvinder oftest indenfor 3-6 måneder men kan hos enkelte blive permanente (mindre end 1%).
Udtagning af muskelbiopsien sker i forbindelse med syning af rotator cuffen ved din kikkertvejledte operation. Vi har i et tidligere forsøg på samme måde udtaget biopsier uden at der er opstået komplikationer herved.
Der er ingen kendte risici ved hverken ultralydsskanning eller MR skanning.
Der udtages *ikke* blodprøver ud over dem der er nødvendige ift. standardbehandlingen.

Udelukkelse fra og afbrydelse af forsøg

Det er kun patienter med rotator cuff læsion, som kan indgå i undersøgelsen.

Hvis der under indlæggelsen eller efterfølgende opstår uforudsete begivenheder, kan det medføre afbrydelse i undersøgelsen. Du vil i så fald blive informeret herom. Forsøget vil blive afbrudt hvis der viser sig uventede bivirkninger ved cellebehandlingen.

Ved standardbehandlingen er der en kendt risiko for at syningen af senen ikke holder. Ved ny skade på senen kan du blive taget ud af forsøget.

Ved eventuel afbrydelse vil behandlingen fortsætte på vanlig vis efter afdelingens normale procedurer.

Oplysninger om økonomiske forhold

Projektet er iværksat af afdelingens idrætssektor, professor, overlæge Lars Henrik Frich.

Undersøgelsen er finansieret af de deltagende afdelinger på Sygehus Sønderjylland (SHS) samt af eksterne midler, der vil blive rejst ud fra en godkendt protokol.

Midlerne vil blive brugt til at betale løn til forsker og projektsygeplejerske, til at købe isolationssæt og betale laboratorieudgifter.

Finansieringen vil blive administreret af forskningsorganisationen på Sygehus Sønderjylland

Behandling af personoplysninger

Databeskyttelsesloven og databeskyttelsesforordningen overholdes i alle projektets faser og projektet er registeret i Region Syddanmarks interne fortegnelse. Behandling af journaldata som indsamles som led i undersøgelsen vil i alle tilfælde anonymiseres før videre bearbejdning. Dette indebærer at personfølsomme oplysninger ikke vil kunne relateres til din person eller dit CPR-nummer.

Da du har et behandlingsforløb på grund af rotator cuff læsion, vil skanningsresultater også indgå i din journal. Disse oplysninger vil vi hente for at vurdere resultatet af behandlingen og kontrollere forsøget. Ved dit samtykke giver du den forsøgsansvarlige, sponsor og dennes repræsentant direkte adgang til relevante helbredsoplysninger i journalen for at kunne gennemføre, overvåge og kontrollere forsøget i hele forsøgsperioden.

Adgang til forsøgsresultater

Informationer fra dette studie vil blive gjort bredt tilgængelig for forskere, læger og videnskabsfolk med henblik på en dybere forståelse af skadens følgevirkninger og med henblik på at finde de rigtige behandlingsmetoder til rotator cuff læsion.

Resultaterne søges offentliggjort i løbet af 2025. Der tilstræbes at offentliggøre ved et internationalt tidsskrift og desuden ved kongresser for forskere, herunder Skandinavien Sports Medicin Kongres, Dansk Ortopædisk Selskab som foredrag samt videnskabelige selskaber i Europa/USA.

Undersøgelsen forventes afsluttet om 3 år.

Vi håber, at du med denne information har fået tilstrækkeligt indblik i, hvad det vil sige at deltage i forsøget. Du velkommen til at kontakte os, hvis du har brug for flere oplysninger.

Med venlig hilsen

Lars Henrik Frich
Professor, Ph.d.
Forsøgsansvarlig
Ortopædkirurgisk afdeling
Sygehus Sønderjylland
Tlf. +45 2027 4119
Email: lars.henrik.frich@rsyd.dk
